# Temporal dynamics of diffusion kurtosis imaging parallels astroglial GFAP expression in a non-traumatic spinal cord injury animal model of ischemia–reperfusion

**DOI:** 10.3389/fneur.2026.1776453

**Published:** 2026-04-24

**Authors:** Li Wang, Xiaoyu Li, Lanbo Wang, Daowei Li

**Affiliations:** 1 Department of Radiology, The People’s Hospital of China Medical University, Shenyang, China; 2 Department of Radiology, Shengjing Hospital of China Medical University, Shenyang, China

**Keywords:** astroglial response, diffusion kurtosis imaging, GFAP, ischemia–reperfusion, rabbit, spinal cord

## Abstract

**Objectives:**

Spinal cord ischemia–reperfusion (I/R) injury triggers rapid secondary degeneration, yet sensitive noninvasive markers that reflect early astroglial and microstructural alterations remain scarce. This study aimed to delineate the temporal changes in GFAP immunoreactivity as an indicator of astroglial response following spinal cord I/R and to determine how these pathological dynamics correspond to diffusion kurtosis imaging (DKI) metrics in a rabbit model.

**Methods:**

Forty-five male New Zealand White rabbits were randomized into a Sham group or four I/R groups with reperfusion durations of 6 h, 24 h, 72 h, and 7 d (*n* = 9/group). DKI (*b* = 0, 1,000, 2000 s/mm^2^) was performed on a 3.0 T scanner to obtain fractional anisotropy (FA), mean diffusivity (MD), and mean kurtosis (MK) maps. Motor function was assessed using the modified Tarlov score. Histopathology, GFAP immunohistochemistry with optical density (OD), and Western blot analysis of L3–L5 segments were performed immediately after imaging. Group comparisons were conducted using ANOVA, and correlations between DKI parameters, GFAP expression, and Tarlov scores were evaluated using Pearson analyses.

**Results:**

Following I/R, GFAP immunoreactivity increased progressively and reached its highest mean value at 72 h, and this temporal pattern was confirmed by Western blot analysis (*p* < 0.001 vs. Sham). This peak coincided with the most pronounced reductions in FA and MK (both *p* < 0.01) and the maximal increase in MD. FA showed a moderate negative correlation with GFAP expression (*r* = −0.547, *p* < 0.01) and with motor function, while MK demonstrated a comparatively weaker negative association. MD correlated weakly and positively with GFAP. Functional deficits paralleled imaging and pathological deterioration, with partial improvement from 72 h and further recovery by 7 d, although not to baseline.

**Conclusion:**

GFAP immunoreactivity after spinal cord I/R follows a well-defined temporal trajectory that closely parallels DKI-derived microstructural alterations, supporting the utility of DKI parameters as noninvasive biomarkers that reflect astroglial response dynamics during early spinal cord I/R injury.

## 1 Introduction

Spinal cord injury (SCI) is devastating primarily because the initial insult is quickly followed by a secondary cascade that exacerbates tissue loss (1, 2). Within hours, ischemia, oxidative stress, excitotoxicity, and inflammation converge, disrupting neuronal and glial homeostasis (3). These processes often determine long-term neurological outcomes more than the initial mechanical injury itself (4). Spinal cord I/R injury represents a distinct clinical scenario within this continuum: its onset is abrupt, the early pathological changes are intense, and the temporal evolution is highly structured (2, 5). Spinal cord I/R represents a clinically relevant and biologically well-defined form of acute SCI, in which secondary degeneration evolves rapidly after reperfusion. Moreover, the highly organized microstructural architecture of the spinal cord makes it a meaningful substrate for diffusion-based evaluation of tissue disruption, even when considering the technical challenges posed by its relatively small size. These characteristics make the I/R model a valuable platform for studying the biological inflection points that shape early SCI progression (1, 2).

Astrocytes play a central role in the response to I/R injury (3, 4). As injury signals accumulate, astrocytes undergo hypertrophy, reorganize their cytoskeleton, and upregulate intermediate filaments such as glial fibrillary acidic protein (GFAP) (5). Early astrocytic activation can be beneficial, helping to modulate excitotoxicity, stabilize the blood–spinal cord barrier, and contain inflammation (6). However, over time, this response shifts toward matrix deposition and structural containment. Astroglial response contributes to the formation of the glial scar—a physical and biochemical barrier that stabilizes the lesion core but also inhibits axonal regeneration (7). Notably, this transition from a supportive to a restrictive phenotype does not occur instantaneously but unfolds over a definable period that may offer therapeutic opportunities (8, 9).

While GFAP expression is widely used as a marker of astroglial response, linking these cellular changes to *in vivo* microstructural alterations requires an imaging modality sensitive to complex tissue organization (8–10). Diffusion kurtosis imaging (DKI) extends beyond traditional diffusion tensor imaging by characterizing non-Gaussian water diffusion (10, 11). This allows DKI to capture scattering effects produced by axonal fragmentation, edema, and glial hypertrophy. Previous studies have demonstrated the value of DKI in several spinal cord disorders, but its application to the acute phase of I/R injury, particularly when paired with time-matched pathological validation, remains limited (12).

This study thus aimed to determine whether DKI metrics could serve as sensitive, noninvasive indicators of early astroglial response, as reflected by GFAP immunoreactivity, in spinal cord I/R. Defining this relationship may help identify biologically meaningful windows for intervention and provide imaging biomarkers capable of guiding future therapeutic strategies.

## 2 Materials and methods

### 2.1 Animals and ethical approval

All experimental procedures complied with institutional and national regulations for animal research and adhered to the ARRIVE 2.0 guidelines (13). The study protocol was reviewed and approved by the Institutional Animal Care and Use Committee of Shengjing Hospital, China Medical University (approval No. 2024PS145K).

**Figure 1:**
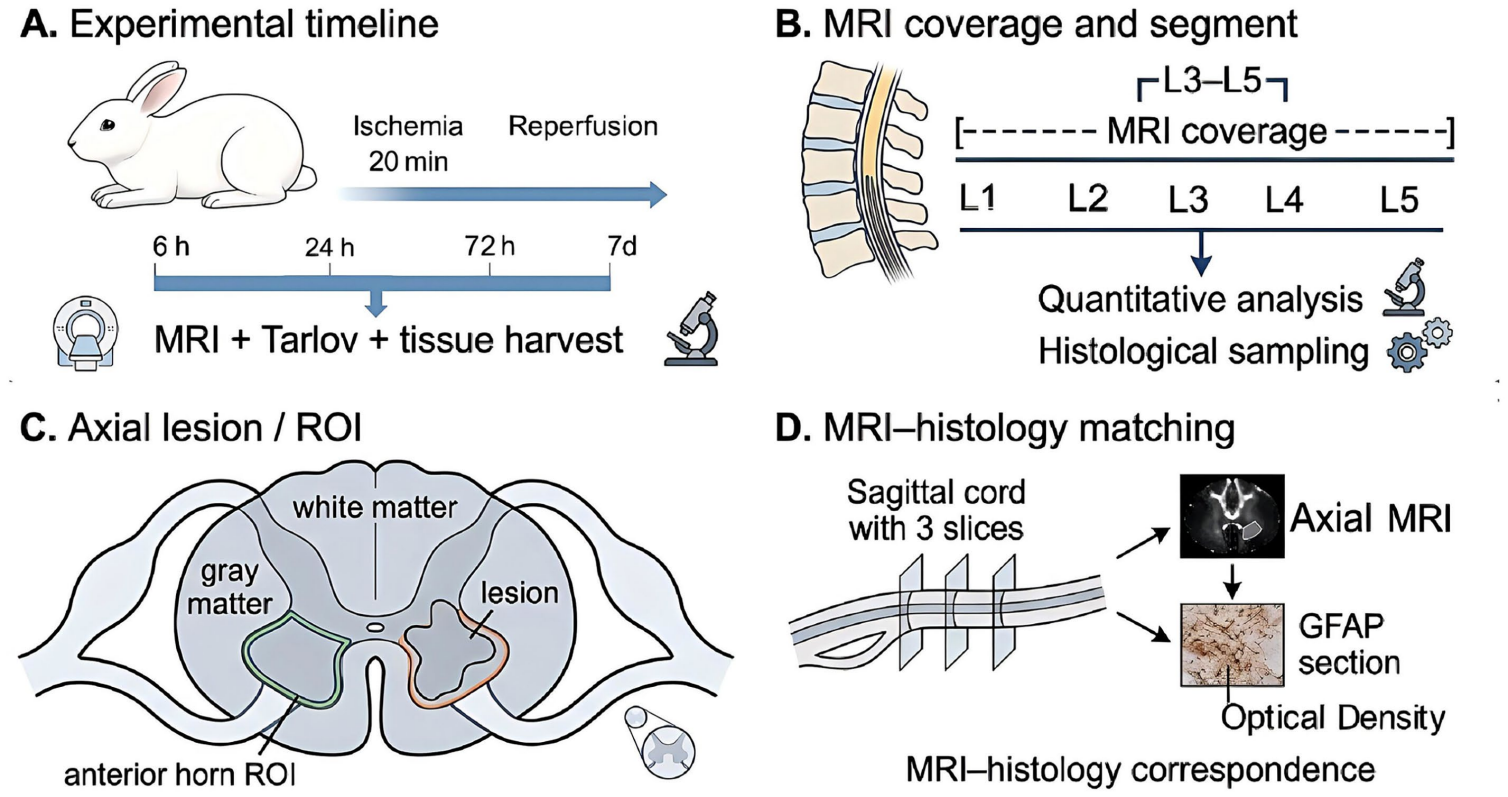
Experimental design, MRI coverage, schematic lesion-related region, and schematic correspondence between MRI slices and histological sampling in a rabbit spinal cord I/R model. **(A)** Experimental timeline. Rabbits underwent sham or ischemia–reperfusion (I/R) surgery with 20 min abdominal aortic occlusion, followed by reperfusion for 6 h, 24 h, 72 h, or 7 d. MRI, behavioral assessment, and tissue collection were performed at each time point. **(B)** Schematic sagittal view of the lumbar spinal cord showing MRI coverage and the L3–L5 segment selected for DKI analysis and histological sampling. **(C)** Schematic axial view showing the lesion-related region and ROI placement in the anterior horn and adjacent white matter for DKI analysis. The ROI area was standardized to 2.0 mm^2^. **(D)** Correspondence between axial MRI slices and matched histological sections used for GFAP immunohistochemistry and optical density measurement. Three contiguous slices were analyzed and averaged for each animal. The lesion-related region shown in the schematic is intended for anatomical illustration only and does not represent a quantitative measurement of lesion volume.

A total of 45 male New Zealand White rabbits (2.5–3.0 kg) were housed in a controlled environment with fixed temperature, humidity, and a 12-h light–dark cycle. The animals were randomly assigned to five groups (n = 9/group): a Sham-operated group and four I/R groups, which represented distinct reperfusion intervals (6 h, 24 h, 72 h, and 7 d) (2, 14, 15). To minimize observer bias, investigators responsible for behavioral scoring, MRI analysis, and histopathology were blinded to the group allocations. The experimental timeline is shown in Figure 1A.

### 2.2 Induction of I/R injury

A reproducible spinal cord ischemia model was generated via transient infrarenal aortic occlusion (14, 15). After pentobarbital anesthesia (30 mg/kg IV), a midline laparotomy exposed the abdominal aorta, which was clamped for 20 min (14). This duration reliably induces lumbar cord ischemia without systemic instability. Sham animals underwent the same exposure but without arterial occlusion. Body temperature was maintained at 37 °C throughout the procedure. The reperfusion period was defined as the interval between clamp release and imaging/tissue collection (6 h, 24 h, 72 h, and 7 d), in accordance with the protocol described in our updated References (2, 14, 15).

### 2.3 MRI acquisition and DKI processing

MRI scans were performed on a 3.0 T clinical system equipped with a dedicated small-animal coil. Rabbits were positioned prone within a custom-designed cradle to stabilize the spine and minimize motion during acquisition. In addition, the torso and limbs were gently secured to maintain a stable position throughout scanning. All imaging data were inspected for artifacts, and scans were deemed adequate for quantitative analysis if no significant distortion was present. MRI coverage and the L3–L5 segment selected for analysis are shown in Figure 1B.

T2-weighted images were acquired using a two-dimensional fast spin-echo sequence to assess spinal cord morphology and edema. The scanning parameters were as follows: repetition time (TR) = 3,000 ms, echo time (TE) = 100 ms, field of view (FOV) = 80 × 40 mm^2^, matrix size = 320 × 160, slice thickness = 1.0 mm, and echo train length = 12. The total acquisition time was approximately 5 min. Diffusion kurtosis imaging (DKI) was acquired using a spin-echo echo-planar imaging sequence with the following parameters: *b*-values of 0, 1,000, and 2000 s/mm^2^; 30 diffusion directions; in-plane resolution of 0.39 × 0.39 mm^2^; and slice thickness of 1 mm. The total acquisition time was approximately 25 min.

The raw diffusion data were corrected for eddy current and geometric distortions (16). DKI data were processed using dedicated software on a Philips Extended Workspace (EWS) workstation. Regions of interest (ROIs) were manually drawn on axial FA maps at the L3–L5 level, including the anterior horn gray matter and adjacent white matter, with values averaged over three consecutive slices for each animal (17). The lesion-related region and ROI placement are shown schematically in Figure 1C. For each animal, three contiguous slices were selected and averaged to yield representative values. ROI placement was independently performed by two experienced neuroradiologists, and any discrepancy greater than 10% was resolved by joint review and consensus with a senior neuroradiologist. All ROIs were circular (2.0 mm^2^, ~13–15 voxels) and manually delineated. Measurements were repeated three times at the same location, and mean values were used for analysis to minimize contamination from adjacent tissues and partial volume effects.

### 2.4 Behavioral assessment

Hindlimb motor function was evaluated using the modified Tarlov score (0–5), a semiquantitative measure sensitive to early neurological deterioration and partial recovery in rabbit I/R models (18, 19). Baseline scores were recorded prior to surgery, and postoperative assessments were performed at each designated reperfusion time point by blinded observers.

### 2.5 Histology and immunohistochemistry

Immediately after MRI scanning, animals were perfused transcardially. L3–L5 spinal cord segments were harvested, fixed in paraformaldehyde, embedded in paraffin, and sectioned for histological and immunohistochemical analysis. The correspondence between the analyzed MRI slices and matched histological sections is shown in Figure 1D.

GFAP immunohistochemistry was performed using a standard horseradish peroxidase (HRP) detection system. Three nonoverlapping regions in the anterior horn were selected for quantitative analysis. To ensure the objectivity and reproducibility of the results, OD analysis was employed instead of manual cell counting. Given that acute I/R injury often leads to cellular edema and indistinct cell boundaries, OD quantification provides a more reliable measure of total GFAP protein expression. The OD was calculated using ImageJ software (National Institutes of Health, USA), with the background signal subtracted to ensure accuracy (20). These quantitative values were used to assess changes in GFAP immunoreactivity for group comparisons and correlation with DKI parameters.

Given the known limitations of GFAP as a lineage-specific marker, the present analysis focused on quantitative assessment of GFAP immunoreactivity rather than morphological identification of specific astrocyte subtypes.

### 2.6 Western blot analysis

Frozen L3–L5 segments were homogenized for total protein extraction. Equal amounts of protein were separated by SDS–PAGE and transferred to PVDF membranes (21). Membranes were incubated with antibodies against GFAP and β-actin, the latter serving as the loading control. Band intensities were quantified using densitometric analysis and normalized to β-actin to account for variations in sample loading.

### 2.7 Statistical analysis

All statistical analyses were performed using standard software. The data distribution was checked prior to analysis. Intergroup comparisons were performed using one-way ANOVA or the Kruskal–Wallis test as appropriate. One-way ANOVA was used to analyze serial Tarlov scores. Pearson correlation coefficients were calculated to assess relationships between DKI parameters (FA, MK, MD), GFAP expression (OD and Western blot values), and motor outcomes. Statistical significance was defined as *p* < 0.05.

## 3 Results

### 3.1 General observations

All 45 rabbits survived the surgical procedures and completed the experimental protocol without intraoperative or perioperative mortality. Postoperative recovery was uneventful, and no animal developed complications severe enough to interfere with behavioral testing, MRI acquisition, or tissue processing. Physiological stability was maintained across all groups.

### 3.2 Motor performance and histopathology

**Figure 2:**
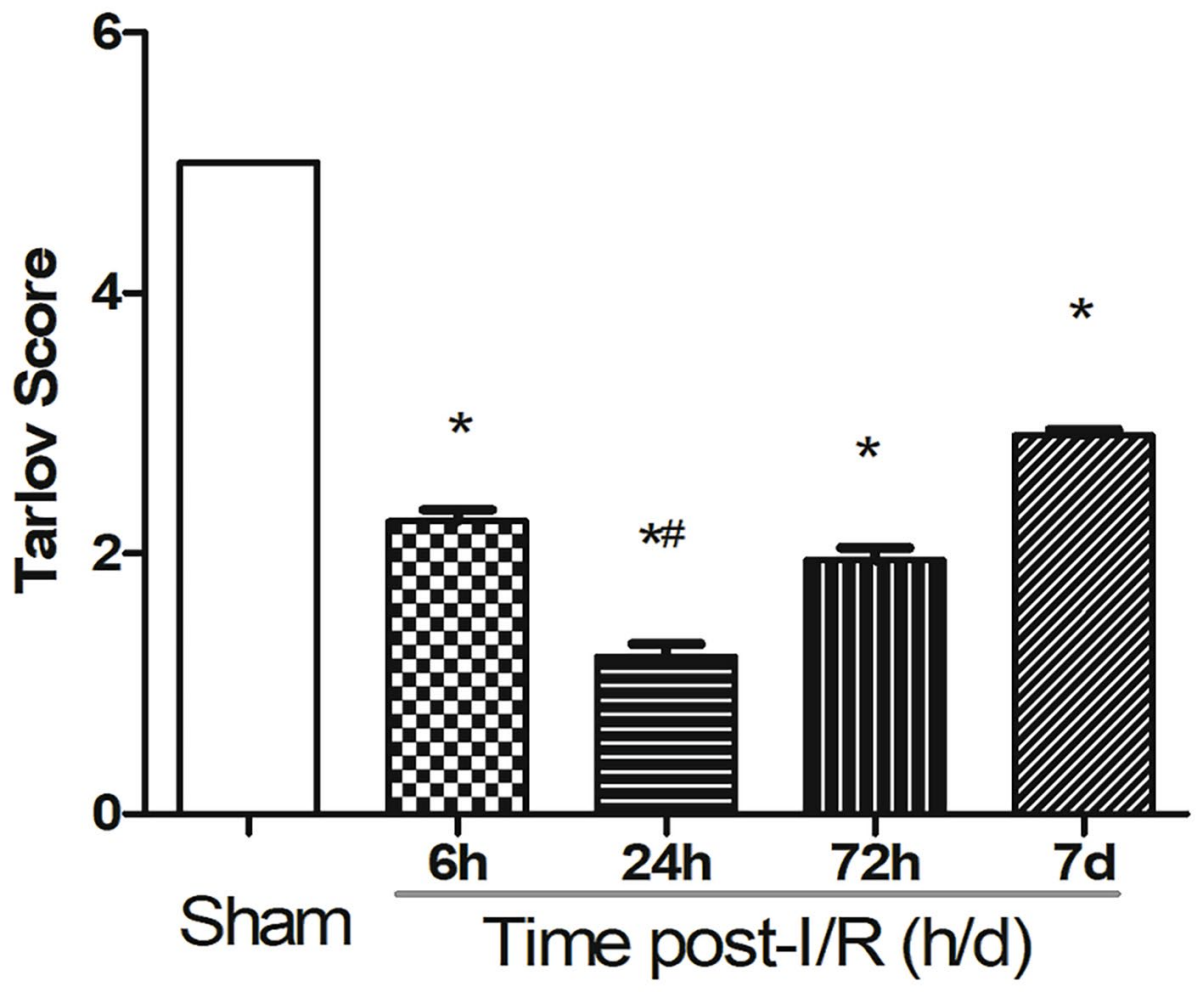
Tarlov scores at different reperfusion time points in the rabbit spinal cord ischemia–reperfusion model. Motor function declined rapidly after reperfusion and partially recovered by 72 h. Data are presented as mean ± SEM. Group comparisons were performed using ANOVA followed by Tukey–Kramer *post hoc* tests. ^*^p < 0.01 vs. Sham; ^#^*p* < 0.05 vs. other post-I/R groups.

Motor function deteriorated rapidly following reperfusion. Tarlov scores showed a significant decline as early as 6 h, reaching their lowest values at 24 h, and exhibited partial improvement by 72 h. These temporal fluctuations were statistically significant across groups [*F*(4,40) = 58.72, *p* < 0.001]. While several animals demonstrated functional recovery by day 7, none achieved a return to pre-injury baseline levels. Group-wise Tarlov score differences are illustrated in Figure 2.

Histopathological findings mirrored the behavioral results. Mild tissue edema and early neuronal alterations were observed at 6 h. By 24 h, inflammatory cell infiltration and focal necrosis became more prominent. The 72 h time point displayed the most severe morphological disruptions, characterized by widespread neuronal loss, axonal fragmentation, myelin sheath disorganization, and cavitation. At 7 d, early glial cell accumulation and extracellular matrix deposition appeared around the lesion core, suggesting the onset of glial scar formation.

### 3.3 Time course of DKI alterations

DKI parameters exhibited dynamic changes that closely followed the evolving microstructural injury (Table 1). Both FA and MK decreased progressively after reperfusion, with the sharpest declines occurring at 72 h (FA: 0.332 ± 0.012; MK: 0.656 ± 0.044). MD increased steadily over the same period and reached its highest value at 72 h, consistent with enhanced extracellular water and reduced diffusion restriction. None of the DKI parameters returned to near-Sham levels at 7 d, despite slight improvements.

**Table 1:** Means (SD) of DKI parameter values (FA, MD, MK) in each group (*n* = 9).

DKI parameter value	Sham	Time post-I/R				*F* values
		6 h	24 h	72 h	7 d	
MD	1.242 ± 0.017	1.25 ± 0.05	1.523 ± 0.027	1.622 ± 0.009*	1.592 ± 0.044*	33.77
FA	0.684 ± 0.021	0.53 ± 0.05	0.523 ± 0.071*	0.332 ± 0.012**#	0.343 ± 0.022*	45.21
MK	1.425 ± 0.076	1.26 ± 0.03	0.814 ± 0.069*	0.656 ± 0.044**#	0.735 ± 0.221*	38.64

Values are given as mean ± SD (*n* = 9 per group). One-way ANOVA with Tukey’s *post hoc* test.

*p < 0.05 vs. Sham; **p < 0.01 vs. Sham; #*p* < 0.05 vs. other post-I/R groups.

FA (unitless, 0–1), fractional anisotropy; MD (μm^2^/ms), mean diffusivity; MK (unitless, >0), mean kurtosis.

**Figure 3:**
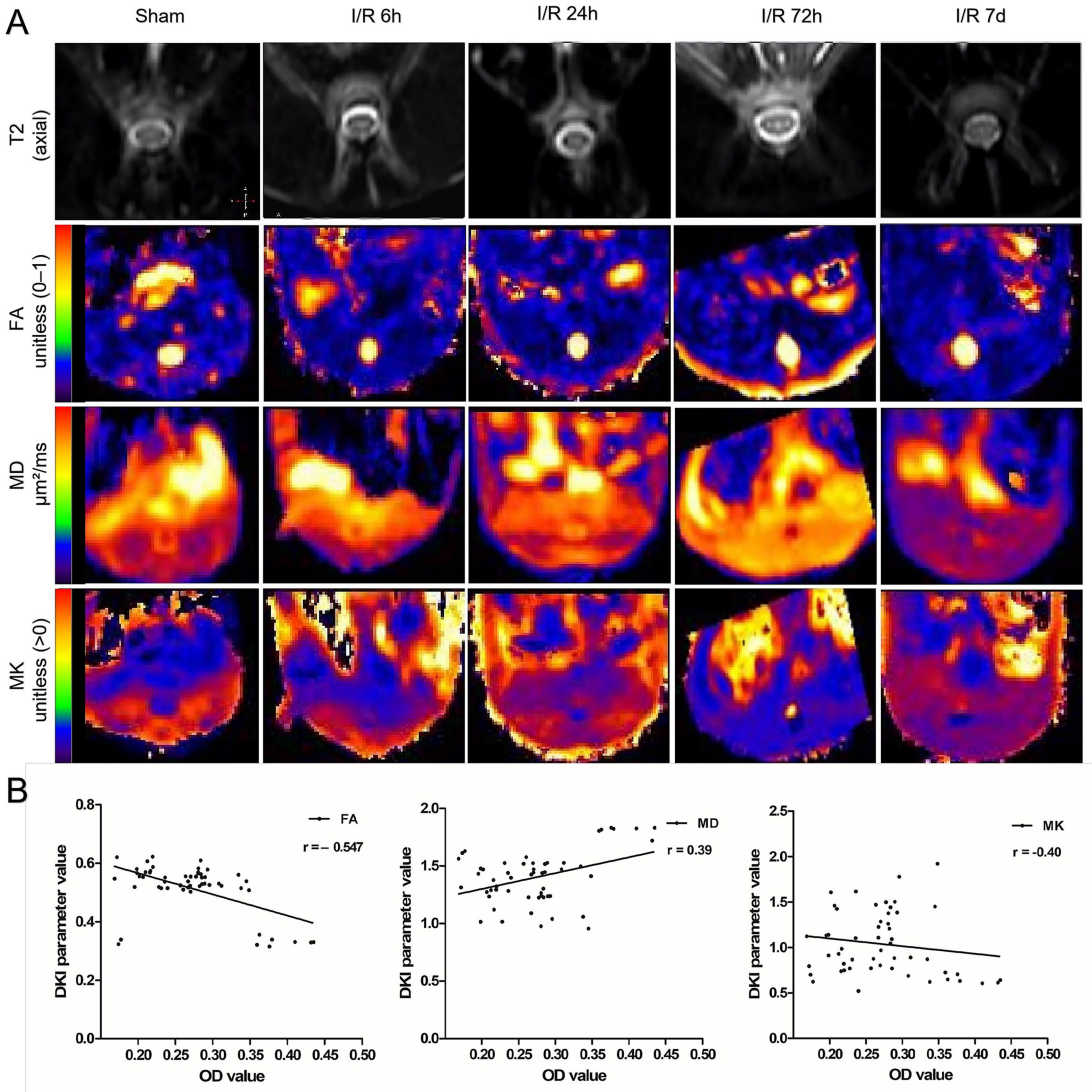
Temporal trends of DKI parameters after spinal cord ischemia–reperfusion. **(A)** representative axial T2-weighted images and DKI parameter maps (FA, MD, MK), obtained at different reperfusion time points. Microstructural disruption is characterized by decreased FA and MK values and increased MD values, with the most pronounced alterations observed at 72 h after reperfusion. **(B)** Correlation analysis between DKI parameters (FA, MD, MK) and OD values. Scatter plots with linear regression lines show the relationship between DKI metrics and GFAP OD. FA showed a moderate negative correlation with GFAP OD (*r* = −0.547, *p* < 0.01), MK showed a similar but less pronounced negative correlation. MD showed a weak positive correlation with GFAP. FA (unitless, 0–1), fractional anisotropy; MD (μm^2^/ms), mean diffusivity; MK (unitless, >0), mean kurtosis.

These alterations were readily observable on parametric maps. At 72 h, FA maps demonstrated a marked loss of anisotropy with blurred delineation of white matter tracts, while MK maps revealed substantial reductions in tissue complexity. Representative images are shown in Figure 3A.

### 3.4 Temporal changes in GFAP expression

#### 3.4.1 Immunohistochemistry

**Figure 4:**
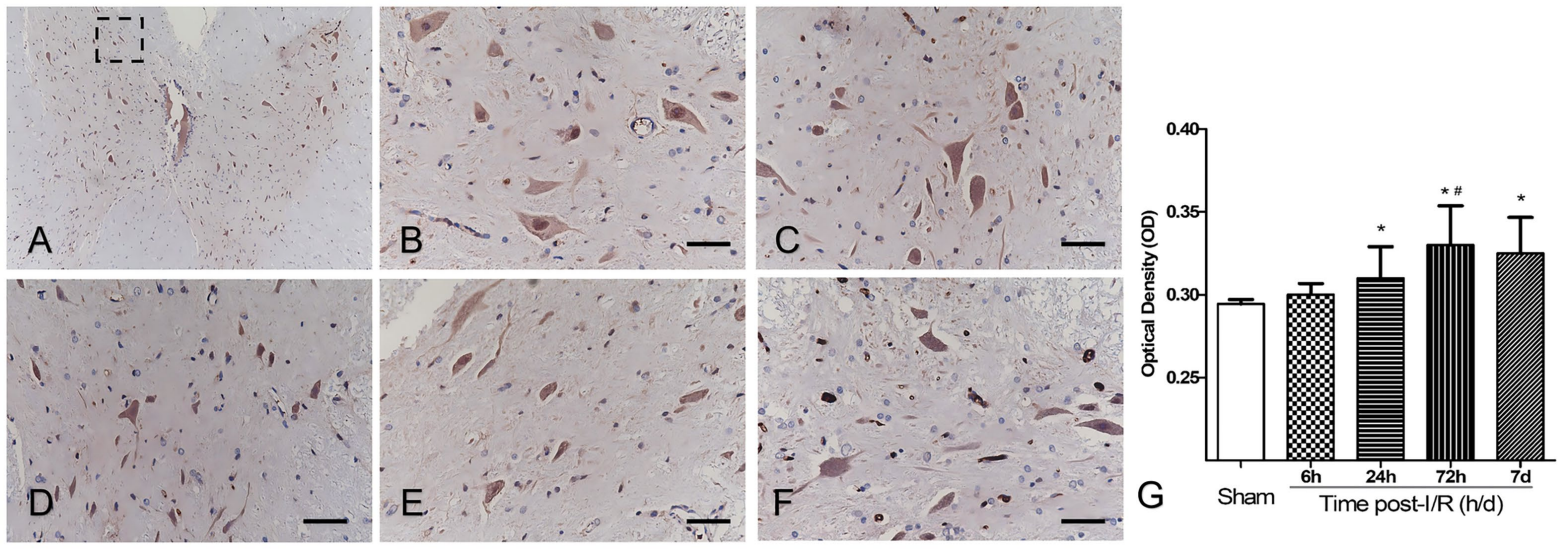
GFAP immunohistochemistry in the anterior horn after spinal cord ischemia–reperfusion (I/R). **(A)** Low-magnification image indicating the anterior horn ROI (dashed box) used for OD quantification. **(B–F)** Representative GFAP immunostaining images at baseline (Sham) and at different reperfusion time points (I/R 6 h, 24 h, 72 h, and 7 d). Scale bars = 20 μm. **(G)** Quantification of anterior horn GFAP immunoreactivity using OD analysis. OD values increased after reperfusion and reached their highest mean value at 72 h. Data are presented as mean ± SEM. One-way ANOVA followed by Tukey’s *post hoc* test. ^*^p < 0.01 vs. Sham (24 h, 72 h, and 7 d); ^#^*p* < 0.05 vs. the other post-I/R groups (6 h, 24 h, and 7 d).

In Sham-operated animals, GFAP-positive cells were sparsely distributed with thin, well-organized processes. GFAP expression showed a mild upward trend at 6 h without reaching statistical significance and increased significantly at 24 h and 72 h. The highest mean labeling intensity was observed at 72 h (*p* < 0.01 vs. Sham). Although expression declined slightly at 7 d, it remained significantly elevated compared with Sham controls (*p* < 0.01), indicating sustained elevation of GFAP immunoreactivity (Figures 4A–F). Quantitatively, anterior horn GFAP immunoreactivity (OD) increased after reperfusion and peaked at 72 h (Figure 4G). OD values were significantly higher at 24 h, 72 h, and 7 d compared with Sham controls (all *p* < 0.01 vs. Sham), and the 72 h group was significantly higher than the other post-I/R time points (6 h, 24 h, and 7 d; #*p* < 0.05). Data are presented as mean ± SEM (*n* = 9 per group).

#### 3.4.2 Western blot analysis

**Figure 5:**
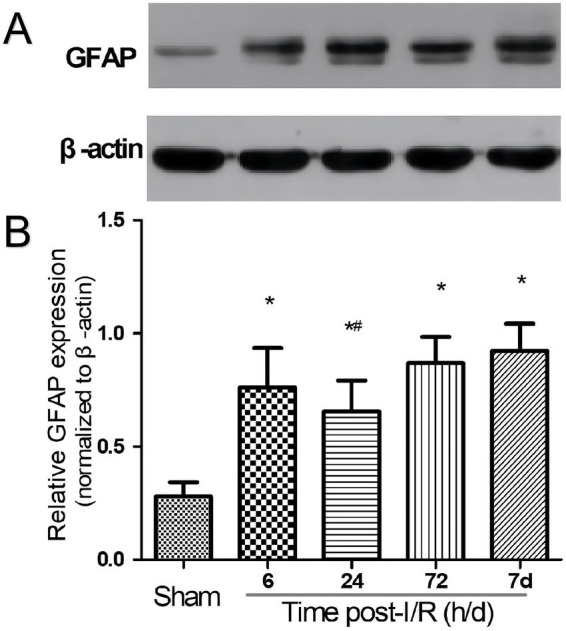
Western blot analysis of GFAP expression. **(A)** Representative western blot bands of GFAP and β-actin across sham and I/R groups. **(B)** Quantification of normalized GFAP/β-actin ratios. GFAP expression increased after I/R and remained elevated from 24 h to 7 d. Data are presented as mean ± SEM. *p < 0.05 vs. Sham; #*p* < 0.05 vs. other post-I/R groups, as indicated.

Western blot quantification corroborated the immunohistochemical results. GFAP protein levels increased after I/R and remained elevated at 24 h, 72 h, and 7 d, with no clear increase beyond the 72 h level (*p* < 0.05 vs. Sham). By 7 d, GFAP levels remained above Sham values despite a modest decline from the peak period. Representative blots and quantification curves are shown in Figure 5.

Together, the immunohistochemical and Western blot data showed a similar temporal pattern, indicating that GFAP immunoreactivity follows a reproducible trajectory during the acute phase of I/R injury, with maximal reactivity occurring around 72 h.

### 3.5 Correlation between DKI parameters and OD values

FA demonstrated a moderate negative correlation with OD values (*r* = −0.547, *p* < 0.01), indicating that reductions in anisotropy closely track the degree of GFAP immunoreactivity. MK showed a similar but less pronounced negative correlation. MD showed a weak positive correlation with GFAP (*r* = 0.39), which did not reach statistical significance (*p* > 0.05) (Figure 3B).

## 4 Discussion

In this study, we systematically characterized the early temporal evolution of GFAP immunoreactivity as a marker of astroglial response, diffusion-based microstructural alterations, and functional impairment during the first week after spinal cord I/R. Across imaging, histological, and behavioral domains, a convergent pattern emerged, with the most pronounced changes occurring around 72 h after reperfusion. Although neurological deficits manifested rapidly and reached substantial severity within the first 24 h, GFAP immunoreactivity and DKI-derived microstructural disruption continued to intensify, culminating at approximately 72 h. This temporal convergence highlights a critical biological phase during which cellular responses and tissue-level alterations are most tightly coupled.

The 72-h mark has long been considered a critical biological inflection point in various central nervous system (CNS) injury models (22–24). During this phase, astrocytes transition from their initial protective role to a more fibrotic, scar-oriented phenotype, contributing to scar formation and tissue remodeling (25). Our findings reflect this shift in GFAP-associated astroglial response. The progressive increase in GFAP expression, with the highest mean level observed at 72 h, aligns with the steepest declines in FA and MK, suggesting a strong relationship between GFAP immunoreactivity and microstructural deterioration (26). This temporal alignment is crucial as it defines a critical phase where interventions targeting glial behavior could potentially be most effective (27, 28). The largest increase in MD further supports the notion of increased extracellular water and the loss of tissue integrity, consistent with the pathological observations of edema and cellular breakdown.

Our observation of GFAP expression patterns under acute ischemic stress aligns with the fundamental principles of reactive gliosis (2–4, 15), although the rapid onset of edema in this rabbit model resulted in somewhat altered glial morphology (10). To account for these pathological complexities and ensure objective quantification, we utilized OD analysis, which provides a more robust measure of protein expression levels than traditional morphological identification. Notably, the localized OD variations in the anterior horn corresponded with total protein fluctuations measured by Western blot, reinforcing GFAP as a reliable biomarker within this ischemic window and establishing a firm foundation for subsequent imaging-pathology correlation.

Among the DKI metrics examined, FA showed a moderate correlation with both GFAP expression and motor performance, suggesting that anisotropy loss is particularly sensitive to the combined effects of axonal disruption and astroglial remodeling. Reduced FA likely reflects not only axonal fragmentation and demyelination, but also the cumulative impact of glial hypertrophy, cytoskeletal reorganization, and extracellular matrix expansion, all of which contributes to altered diffusion directionality (29, 30). In contrast, MK followed a similar temporal trajectory but exhibited a more limited dynamic range, potentially due to the competing influences of cellular swelling, edema, and evolving tissue heterogeneity (26). MD changes were more modestly correlated with GFAP expression, consistent with its primary sensitivity to increased extracellular water content and loss of diffusion restriction rather than specific cellular processes (28, 29).

A key strength of this study is the synchronized acquisition of both imaging and pathological data at predefined time points, enabling a direct comparison of DKI-derived changes with molecular alterations. This approach provided a more accurate mapping of GFAP-associated astroglial response alongside structural damage, rather than relying on indirect inferences from either modality alone (27, 31). By identifying a time window where imaging changes and biological processes are most tightly coupled, our study provides valuable insights into the temporal dynamics of spinal cord I/R injury, offering a potential avenue for both experimental and clinical applications (22).

In contrast to our previous feasibility-focused work (2), the current study integrates refined early reperfusion time points with quantitative GFAP assessment (IHC optical density and Western blot) to validate DKI changes. The concurrence of peak GFAP expression and maximal DKI alterations around 72 h supports an association between GFAP immunoreactivity and microstructural disruption in the early post-reperfusion period, providing additional translational context for therapeutic timing.

However, several limitations should be considered. First, the glial response was evaluated mainly by GFAP expression, which reflects only one aspect of reactive gliosis. Additional markers for microglia, oligodendrocytes, and extracellular matrix components would provide a more comprehensive assessment of the glial response to I/R injury (32, 33). Second, although ROI-based sampling enabled targeted analysis, it may not fully reflect the spatial heterogeneity of injury, particularly in focal spinal cord ischemia. Third, lesion volume was not quantitatively measured in the present study. Therefore, the potential contribution of inter-animal differences in lesion burden to variability in DKI and GFAP-related parameters could not be determined. Future studies incorporating quantitative lesion volumetry are needed to address this issue. Fourth, partial volume effects cannot be completely excluded in diffusion-based imaging because of the small size of the spinal cord. Although we sought to reduce this effect by using axial acquisitions, relatively high in-plane resolution, and carefully restricted ROIs, some voxel-level tissue mixing may still have influenced the measured parameters (34). Fifth, although GFAP is a well-established marker of reactive astrogliosis, it does not capture the full range of molecular changes associated with astroglial activation and is not entirely specific to mature astrocytes under pathological conditions (35). Accordingly, the present findings should be interpreted as reflecting changes in GFAP immunoreactivity rather than definitive changes in mature astrocyte number. The moderate correlation between FA and GFAP expression (*r* = −0.54) further supports the multifactorial nature of spinal cord injury. Future studies incorporating markers such as Vimentin, Nestin, S100B, Iba-1, and MBP may help further define the relationship between DKI metrics and specific pathological changes.

From a translational perspective, the identification of a reproducible window of heightened GFAP immunoreactivity and microstructural instability is crucial for guiding therapeutic interventions. Targeting specific pathways involved in astroglial reactivity—such as STAT3 signaling or chondroitin sulfate proteoglycan (CSPG) deposition—during this critical window could improve therapeutic outcomes (24, 25, 36). Notably, the clinical relevance of “ultra-early” MRI (typically within 24–72 h post-injury) as a prognostic tool has been increasingly recognized, particularly in identifying patients at risk for permanent neurological deficits (12). Our results complement these clinical observations by demonstrating that DKI can detect subtle microstructural shifts-specifically those paralleling GFAP immunoreactivity-within the same ultra-early window (37). Therefore, DKI parameters, particularly FA and MK, offer a noninvasive means of monitoring these processes *in vivo*, providing a valuable tool for both preclinical studies and clinical trials (34, 36, 38). These imaging metrics could serve as biomarkers for assessing the efficacy of interventions aimed at modulating astroglial response and mitigating the effects of glial scarring (27, 29).

In summary, our study demonstrates that the temporal dynamics of GFAP immunoreactivity and DKI-derived metrics, particularly FA and MK, are tightly coupled in the early phases of spinal cord I/R injury. These findings highlight the potential of DKI as a tool for identifying therapeutic windows and guiding treatment decisions in spinal cord injury, with further validation needed for clinical application.

## Data Availability

The original contributions presented in the study are included in the article/supplementary material, further inquiries can be directed to the corresponding authors.
